# *Spirulina platensis* Mitigates the Inhibition of Selected miRNAs that Promote Inflammation in HAART-Treated HepG2 Cells

**DOI:** 10.3390/plants12010119

**Published:** 2022-12-26

**Authors:** Thabani Sibiya, Terisha Ghazi, Jivanka Mohan, Savania Nagiah, Anil A. Chuturgoon

**Affiliations:** 1 Department of Medical Biochemistry and Chemical Pathology, School of Laboratory Medicine and Medical Sciences, College of Health Sciences, University of KwaZulu-Natal, Howard College Campus, Durban 4013, South Africa; 2 Department of Human Biology, Medical Programme, Faculty of Health Sciences, Nelson Mandela University Missionvale, Bethelsdorp, Port Elizabeth 6059, South Africa

**Keywords:** highly active antiretroviral therapy (HAART), *Spirulina platensis*, oxidative stress, antioxidant, micro-RNA, inflammation

## Abstract

The introduction of highly active antiretroviral therapy (HAART) in the treatment of HIV/AIDS has recently gained popularity. In addition, the significant role of microRNA expression in HIV pathogenesis cannot be overlooked; hence the need to explore the mechanisms of microRNA expression in the presence of HAART and *Spirulina platensis* (SP) in HepG2 cells. This study investigates the biochemical mechanisms of microRNA expression in HepG2 cells in the presence of HAART, SP, and the potential synergistic effect of HAART–SP. A 3-(4,5-dimethylthiazol-2-yl)-2,5-diphenyltetrazolium bromide (MTT) assay was used to determine cell viability following SP treatment. The cellular redox status was assessed using the quantification of intracellular reactive oxygen species (ROS), lipid peroxidation, and a lactate dehydrogenase (LDH) assay. The fluorometric JC-1 assay was used to determine mitochondrial polarisation. The quantitative polymerase chain reaction (qPCR) was also employed for micro-RNA and gene expressions. The results show that MiR-146a (*p* < 0.0001) and miR-155 (*p* < 0.0001) levels increased in SP-treated cells. However, only miR-146a (*p* < 0.0001) in HAART–SP indicated an increase, while miR-155 (*p* < 0.0001) in HAART–SP treatment indicated a significant decreased expression. Further inflammation analysis revealed that Cox-1 mRNA expression was reduced in SP-treated cells (*p* = 0.4129). However, Cox-1 expression was significantly increased in HAART–SP-treated cells (*p* < 0.0001). The investigation revealed that HepG2 cells exposed to HAART–SP treatment showed a significant decrease in Cox-2 (*p* < 0.0001) expression. mRNA expression also decreased in SP-treated cells (*p* < 0.0001); therefore, SP potentially controls inflammation by regulating microRNA expressions. Moreover, the positive synergistic effect is indicated by normalised intracellular ROS levels (*p* < 0.0001) in the HAART–SP treatment. We hereby recommend further investigation on the synergistic roles of SP and HAART in the expression of microRNA with more focus on inflammatory and oxidative pathways.

## 1. Introduction

HAART is a combination of drugs used to combat human immunodeficiency virus (HIV) that continues to be a global public concern due to its alarming infection rate and mortality rate [1]. Following a recent report from Joint United Nations Programme on HIV/AIDS (UNAIDS) in November 2021, an estimated figure of approximately 37.7 million people globally are living with HIV. It was also reported that approximately 1.5 million new HIV-infected persons were recorded with approximately 680,000 deaths in 2020 [1,2,3,4]. South Africa has one of the highest infection rates; approximately 8.2 million South Africans are living with HIV in the year 2021 [4]. The above statistic could have been worse without the availability of antiretrovirals (ARVs) that have also helped in the lifespan elongation of the persons with HIV-AIDS and reducing the number of people infected with the virus. Globally, approximately 27.5 million HIV-infected persons had access to ARVs in 2020 while approximately 5.6 million infected South Africans accessed ARVs in 2020 [1,4,5].

HAART prolongs HIV-infected patients’ lifespans through the regulation of the viral load and prevention of the associated symptoms from progressing to AIDS. Despite its success, the use of HAART promotes metabolic syndrome through inflammatory pathways, excessive production of reactive oxygen species (ROS), and mitochondrial dysfunction [6,7,8,9,10,11,12]. There are antioxidant agents capable of ameliorating metabolic syndromes; cyanobacteria such as *Spirulina platensis* (SP) have been well documented for this ability [13,14].

SP possesses various medicinal properties that include building the humoral and cellular mechanisms of the immune system when consumed [15]. Interestingly, SP is linked with metabolic syndrome-lowering properties such as hypoglycemia [16], hypolipidemia [17], antihypertension [18]. Studies in some rodent species suggest that SP is mainly useful in the prevention of metabolic syndrome [18]. SP contains bioactive substances such as carotenoids, phenols, chlorophylls, phycocyanin, polyunsaturated fatty acids (PUFAs), glycosides, flavonoids, and alkaloids, according to studies [19,20,21,22,23]. SP contains oxidative stress inhibitors, phycocyanin, and phycocyanobilin [14,24,25]. Phycocyanin is responsible for reducing oxidative stress via the inhibition of NADPH oxidase and suppresses the activation of inflammation [13]. SP also inhibits oxidative stress [13,14,26,27], promotes mitochondrial health [28,29,30,31], and inhibits inflammation [14,32]. Furthermore, it has been found to be useful in the prevention of atherosclerosis [13] and diabetes development [14].

Biological processes such as cell proliferation and apoptosis require small non-coding RNAs called microRNA (miRNA) for gene regulation [33]. MiRNA are approximately 22 nucleotides in length and are generated from long primary miRNA transcripts. The main function of miRNA is to control gene expression at the post-transcriptional level through degrading or repressing target mRNAs [34]. It is estimated that 30% of all human gene expressions are regulated by miRNAs [35]. MiRNAs are important in the coordination of many cellular processes such as regulating apoptosis, proliferation, differentiation, development, and metabolism [36,37,38]. MiRNA plays important regulatory roles in a variety of biological processes including metabolic processes (metabolic integration, insulin resistance, and appetite regulation) [39]. There is evidence supporting the role of miRNAs as an important inflammatory mediator by regulating both adaptive and innate immunity [40].

Oxidative stress results in the dysregulation of signaling pathways associated with metabolism and epigenetics, including microRNAs, which are biomarkers of metabolic disorders. Studies have proven that different sources of oxidative stress change the expression of numerous microRNAs in organs involved in the regulation of glucose and lipid metabolism and endothelium. Dysregulated microRNAs either directly or indirectly affect the expression and activity of molecules of antioxidative signaling pathways, as well as genes of numerous signaling pathways connected with inflammation, insulin sensitivity, and lipid metabolism, thus promoting the progression of metabolic imbalance [41].

Specific miRNAs, such as miR-155 and miR-146a, were initially linked with the inflammatory response by virtue of their potent up-regulation in multiple immune cell lineages by Toll-like receptor ligands, inflammatory cytokines, and specific antigens. However, the increased expression of miR-155 and miR-146a in metabolic syndrome was found to contribute to inflammation-mediated glomerular endothelial injury [42]. Due to the alarmingly increasing number of HIV-infected people and their high dependence on HAART, this study investigates micro-RNA involvement in the inflammation pathway.

## 2. Results

### 2.1. Cell Viability

**Figure 1:**
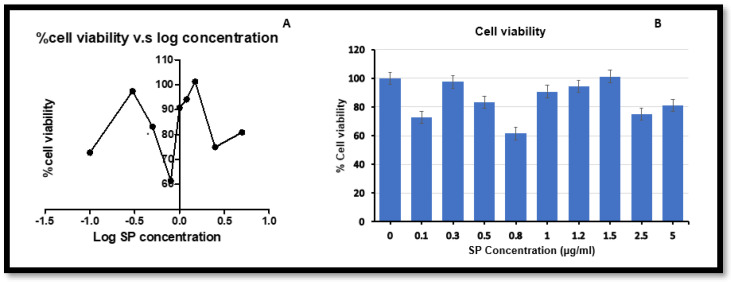
The effects of increased SP treatment concentration on the cell viability in HepG2 cells after 24 h. (**A**) Overall SP increased the cell viability above that of control cells; (**B**) SP concentration of 1.5 µg/mL showed to be more favourable in maintaining cell viability.

The MTT assay was used to determine cell viability and to confirm the suitable concentration for SP treatment; 1.5 µg/mL SP concentration is supported by range from other studies [43]. Figure 1A shows that cell viability mostly increased with increased SP concentrations. Figure 1B indicates that 1.5 µg/mL is more beneficial to HepG2 cell viability. Moreover, an IC_50_ value was calculated using GraphPad Prism 5.0 and was determined to be 11.75 µg/mL for SP in HepG2 cells.

### 2.2. MicroRNA Response

**Figure 2:**
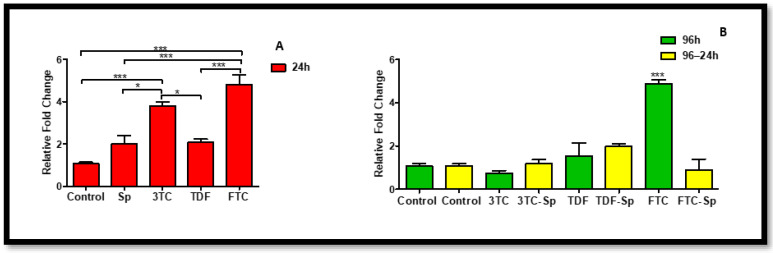
Effects of SP and HAART (3TC, TDF, and FTC) on MiR-146a levels. MiR-146a levels after exposure of HepG2 cells to (**A**) SP and HAART for 24 h, (**B**) HAART (96 h), and HAART (96 h) followed by SP (24 h); * p < 0.05, *** *p* < 0.0001.

**Figure 3:**
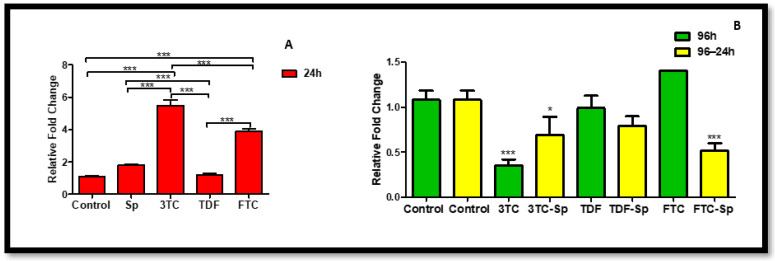
Effects of SP and HAART (3TC, TDF, and FTC) on the miR-155 levels. MiR-155 levels after exposure of HepG2 cells to (**A**) SP and HAART for 24 h, (**B**) HAART (96 h), and HAART (96 h) followed by SP (24 h); * *p* < 0.05, *** *p* < 0.0001.

The main function of miRNA is to control gene expression at the post-transcriptional level through degrading or repressing target mRNAs. MiR-146a levels A: (*p* < 0.0001), B: (*p* < 0.0001) increased in SP- and HAART-treated cells except 3TC. HAART–SP also indicated an increased miR-146a level except FTC-SP B: (*p* < 0.0001). (Figure 2). The miR-155 levels increased in SP- and HAART-treated cells except 3TC and TDF A: (*p* < 0.0001), B: (*p* < 0.0001). HAART–SP-treated cells indicated a significant decrease in miR-146a levels B: (*p* < 0.0001) (Figure 3).

### 2.3. Cyclooxygenase (Cox) Family Response

**Figure 4:**
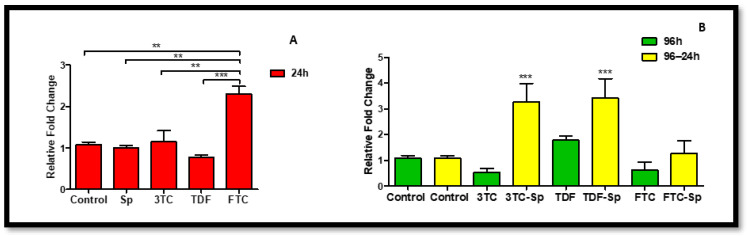
Effects of SP and HAART (3TC, TDF, and FTC) on *Cox-1* mRNA expression. *Cox-1* mRNA expression after exposure of HepG2 cells to (**A**) SP and HAART for 24 h, (**B**) HAART (96 h), and HAART (96 h) followed by SP (24 h); ** *p* < 0.005, *** *p* < 0.0001.

**Figure 5:**
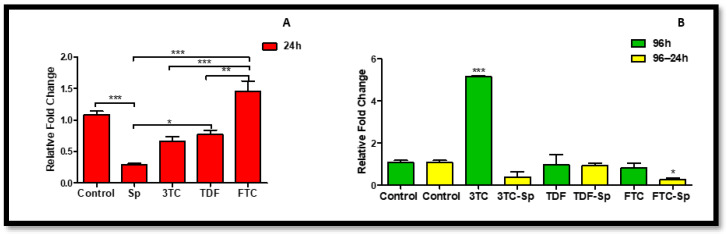
Effects of SP and HAART (3TC, TDF, and FTC) on *Cox-2* mRNA expression. *Cox-2* mRNA expression after exposure of HepG2 cells to (**A**) SP and HAART for 24 h, (**B**) HAART (96 h), and HAART (96 h) followed by SP (24 h); * *p* < 0.05, ** *p* < 0.005, *** *p* < 0.0001.

Cyclooxygenase-2 (Cox-2) is expressed by inflammatory cells, such as macrophages, and can be induced by TNF. Cox-2 is a central link to various inflammatory processes [44]. Cox-2 has been associated with inflammation, whereas the constitutively expressed Cyclooxygenase-1 (Cox-1) is generally considered as a housekeeping enzyme. However, recent evidence suggests that Cox-1 can also be upregulated and may play a prominent role in the brain during neuroinflammation [45]. *Cox-1* mRNA expression was reduced in SP-treated cells and mostly decreased in HAART (except TDF)-treated cells A: (*p* = 0.0003), B: (*p* < 0.0001). However, *Cox-1* expression is significantly increased in HAART–SP treated cells B: (*p* < 0.0001) (Figure 4). *Cox-2* mRNA expression is decreased in SP-treated cells at 24 h exposure A: (*p* < 0.0001), and mostly reduced in HAART (except 3TC)-treated cells after 24 h exposure B: (*p* < 0.0001) (Figure 5). However, cells exposed to HAART–SP treatment showed a significant decrease B: (*p* < 0.0001) (Figure 5).

### 2.4. Jun N-Terminal Kinases (JNK)

**Figure 6:**
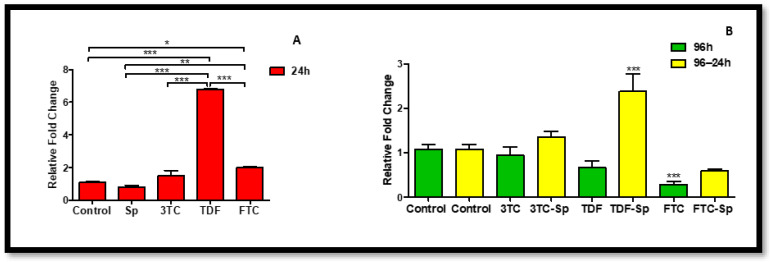
Effects of SP and HAART (3TC, TDF, and FTC) on *JNK* mRNA expression. *JNK* mRNA expression after exposure of HepG2 cells to (**A**) SP and HAART for 24 h, (**B**) HAART (96 h), and HAART (96 h) followed by SP (24 h); * *p* < 0.05, ** *p* < 0.005, *** *p* < 0.0001.

Jun N-terminal kinases (JNK) belong to the superfamily of MAP kinases that are involved in the regulation of cell proliferation, differentiation, and apoptosis [46]. *JNK* mRNA expression decreased in SP- and HAART-treated cells A: (*p* < 0.0001), B: (*p* < 0.0001). HAART–SP-treated cells showed a decrease in the expression of *JNK* mRNA except TDF-SP B: (*p* < 0.0001) (Figure 6).

### 2.5. Assessment of Oxidative Stress

**Figure 7:**
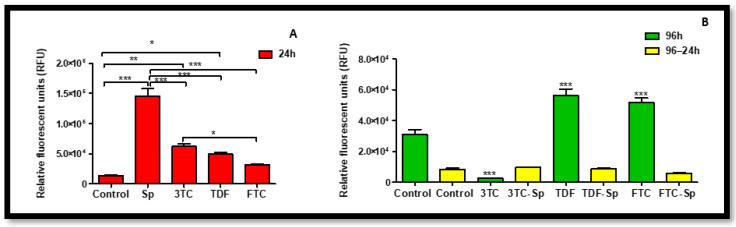
Intracellular ROS levels represented as relative light units (RLU) produced after H_2_DCF-DA staining in HepG2 cells. Intracellular ROS levels after exposure of HepG2 cells to (**A**) SP and HAART for 24 h, (**B**) HAART (96 h), and HAART (96 h) followed by SP (24 h); * *p* < 0.05, ** *p* < 0.005, *** *p <* 0.0001.

Oxidative stress parameters were quantified in HepG2 cells via a H_2_DCF-DA assay. SP-treated cells displayed significant increased levels of intracellular ROS, while HAART also induced a significantly abnormal increase in intracellular ROS following acute and prolonged exposure, with only 3TC (96 h) indicating a significant decrease A: (*p* < 0.0001), B: (*p* < 0.0001). Interestingly, SP managed to reduce access ROS induced by prolonged exposure to HAART, specifically there was a positive synergistic effect, B: (*p* < 0.0001) (Figure 7).

### 2.6. Mitochondrial Stress Responses

**Figure 8:**
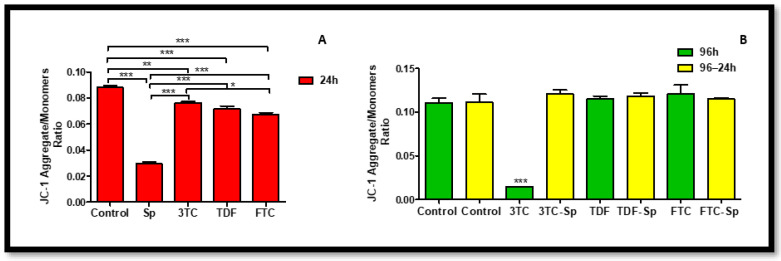
Mitochondrial response. Δmψ represented as a ratio of JC-1 aggregates and JC-1 monomers. The Δmψ after exposure of HepG2 cells to (**A**): SP and HAART for 24 h, (**B**): HAART (96 h), and HAART (96 h) followed by SP (24 h); * *p* < 0.05, ** *p* < 0.005, *** *p* < 0.0001.

**Figure 9:**
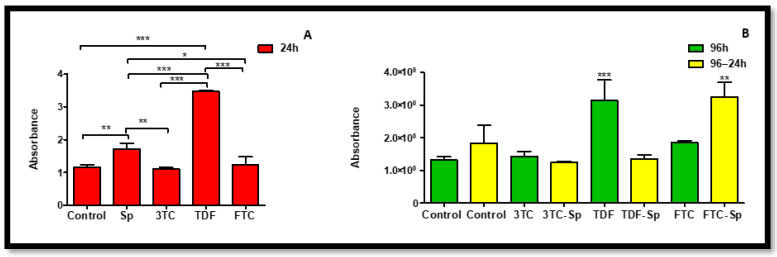
Effects of SP and HAART (3TC, TDF, and FTC) on intracellular LDH levels. Intracellular LDH levels after exposure of HepG2 cells to (**A**) SP and HAART for 24 h, (**B**) HAART (96 h), and HAART (96 h) followed by SP (24 h); * *p* < 0.05, ** *p* < 0.005, *** *p* < 0.0001.

Mitochondrial membrane potential (Δmψ) was measured to determine mitochondrial health and function. The JC-1 assay was used to determine Δmψ. SP and HAART-treated HepG2 cells showed healthy Δψm A: (*p* < 0.0001), B: (*p* < 0.0001), and HAART–SP also showed healthy Δψm results B: (*p* < 0.0001) (Figure 8). Extracellular levels of LDH were quantified using a colorimetric assay to assess the integrity of the cell membrane, since LDH is exclusively found in the cytoplasm and only exits in the cell through damaged membranes [47]. The increase in LDH release suggests increased cell damage [48], and can be an early indicator of increased necrotic cell death. SP and HAART mostly indicated significant elevated LDH levels after acute exposure A: (*p* < 0.0001). However, prolonged exposure of HepG2 cells to HAART followed by acute exposure to SP mostly reduced LDH levels B: (*p* < 0.0001) (Figure 9). Unfavourably, FTC-SP indicated a significant increase (Figure 9).

**Figure 10:**
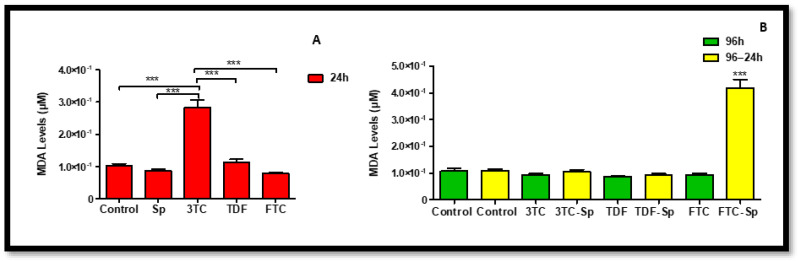
Extracellular MDA levels in SP and HAART (3TC, TDF, and FTC)-treated HepG2 cells. Extracellular MDA levels after exposure of HepG2 cells to (**A**) SP and HAART for 24 h, (**B**) HAART (96 h), and HAART (96 h) followed by SP (24 h); *** *p* < 0.0001.

The MDA levels were quantified in HepG2 cells post chronic exposure to ARVs and acute exposure to SP The MDA levels were significantly decreased in SP-treated cells and significantly increased for 3TC and TDF after acute exposure A: (*p* < 0.0001), while decreased in HAART–SP-treated HepG2 cells compared to the untreated cells B: (*p* < 0.0001), except for FTC-SP, which showed a significant increase (Figure 10).

## 3. Discussion

Studies have shown that the possible therapeutic effects of antioxidants may provide strategies in suppressing oxidative stress- and inflammation-induced comorbidities that emerge with the use of HAART therapy in HIV-infected individuals [11]. The combination of HIV and HAART has been associated with increased oxidative stress and lipid peroxidation [12]. SP is a potent antioxidant [24,25] with anti-inflammatory activities [32], which makes it a potential supplement in the mitigation of oxidative stress induced by HAART-adverse drug reactions. SP can inhibit NADPH oxidase, which is considered as one of the main sources of reactive oxygen species (ROS) and free radicals [32], resulting in reduced oxidative stress [13]. Coincidentally, HAART is known to induce oxidative stress [9,10,49]. SP increased cell viability of HepG2 cell upon acute exposure. The ability of SP was supported by our data from measuring intracellular ROS, where SP managed to bring normalcy (Figure 7). SP-only-treated cells displayed increased levels of intracellular ROS, while HAART induced a significantly abnormal increase (Figure 7). However, SP managed to reduce access ROS induced by prolonged exposure to HAART (Figure 7). SP is rich with antioxidant properties [15,24], and also contains phycocyanin, commonly known for reducing oxidative stress and NADPH oxidase [13]. Hence, the oxidative stress and NADPH oxidase inhibition ability by SP may explain the observed reduction levels of intracellular ROS (Figure 7) following SP exposure in HepG2 cells treated with HAART.

The ETC is responsible for ROS production and complications in this process may result in oxidative stress and depolarisation of the mitochondria, consequently causing a decrease in mitochondrial membrane potential (Δψm) and mitochondrial production of ATP [50]. SP prevented the mitochondrial membrane depolarisation of HepG2 cells, this is demonstrated by the Δψm data (Figure 8). SP- and HAART-treated cells showed healthy Δψm, and HAART–SP also showed healthy Δψm (Figure 8). Studies in vitro showed that SP can scavenge nitric oxide and prevent DNA damage [51], and also can enhance cell nucleus enzyme function and DNA repair synthesis [52]. Moreover, it can enhance mitochondrial health [28,29,30,31]; this agrees with the results observed in this present study.

ROS-induced lipid peroxidation is responsible for oxidative damage and the reduction of cell membrane function [53]. LDH is exclusively found in the cytoplasm and only exits the cell through damaged membranes [47]. The ETC is responsible for ROS production and complications in this process may result in oxidative stress and depolarisation of the mitochondria, consequently causing a decrease in mitochondrial membrane potential and mitochondrial production of ATP [50]. SP and HAART mostly indicated significant elevated LDH levels after acute exposure (Figure 9). However, prolonged exposure of HepG2 cells to HAART followed by acute exposure to SP mostly reduced LDH levels (Figure 9).

Abnormal production of ROS results in the peroxidation of lipids, which produces by-products such as MDA [54]. The MDA levels were significantly decreased in SP-treated cells, while decreased in HAART–SP-treated HepG2 cells compared to the untreated cells (Figure 10). However, there was an increase in MDA levels for FTC-SP (Figure 10), this could be due to the fact that FTC is fluorinated NRTI [55], and fluoride on its own have been linked to oxidative stress, mitochondrial damage, and the alteration of gene expression upon prolonged exposure [56], this could lead to SP requiring more exposure period to mend or bring balance to HepG2 cells that have been exposed to FTC.

The evidence supporting the function of microRNAs (miRNAs) in the control of inflammatory diseases is growing. Dysregulated microRNAs either directly or indirectly affect the expression and activity of molecules of inflammation [41]. The increased expression of miR-155 and miR-146a in metabolic syndromes was found to contribute to inflammation-mediated glomerular endothelial injury [42]. Together, SP and HAART were able to significantly lower miR-155, which may be a sign that the medication is reducing antiinflammation (Figure 3). The main function of miRNA is to control gene expression at the post-transcriptional level through degrading or repressing target mRNAs. SP and HAART together managed to significantly reduce miR-155; this is the sign of reduction of inflammation due to the treatment (Figure 3). MiR-146a levels increased in SP- and HAART- treated cells (Figure 2). However, HAART–SP also indicated an increased miR-146a level (Figure 2), This might be due to a limited exposure time or SP might be using another favourable path to combat inflammation.

Increasing evidence suggests the involvement of microRNA (miR-146a) in the pathogenesis of multiple diseases, including atherosclerosis, bacterial infection, and cancer [57]. MiR-146a levels increased in SP- and HAART-treated cells except 3TC (Figure 2). HAART–SP also indicated an increased miR-146a level except FTC-SP (Figure 2). The miR-155 levels increased in SP- and HAART-treated cells except 3TC and TDF. HAART–SP-treated cells indicated a significant decrease in miR-155 levels (Figure 3). It is noteworthy that the expression of miR-146a in HepG2 cells after exposure to SP and HAART is being tested for the first time in this present study. Studies revealed that miR-146a expression was deceased when c-jun N-terminal kinase (JNK) or nuclear factor (NF)-κB signaling was inhibited, suggesting that there is a correlation between the expression of JNK and miR-146a. Moreover, it has been demonstrated that miR-146a might be useful to inhibit inflammatory activation [57]. In the present study, miR-146a expression decreased in HepG2 cells exposed to HAART, following up with SP.

It has been demonstrated that miR-146a expression levels are significantly lower in lung cancer cells as compared with normal lung cells. Conversely, lung cancer cells have higher levels of cyclooxygenase-2 (Cox-2) protein and mRNA expression [58]. According to Cornett and Lutz [58], the introduction of miR-146a can specifically ablate Cox-2 protein and the biological activity of Cox-2, they proposed that decreased miR-146a expression contributes to the up-regulation and overexpression of Cox-2 in lung cancer cells [58].

Cox-2 is expressed by inflammatory cells, such as macrophages, and can be induced by tumor necrosis factor (TNF). Cox-2 is a central link to various inflammatory processes [44]. Cox-2 has been associated with inflammation, whereas the constitutively expressed cyclooxygenase-1 (Cox-1) is generally considered as a housekeeping enzyme. However, recent evidence suggests that Cox-1 can also be upregulated and may play a prominent role in the brain during neuroinflammation [45]. *Cox-1* mRNA expression was reduced in SP-treated cells and mostly decreased in HAART (except TDF)-treated cells (Figure 4). However, Cox-1 expression is significantly increased in HAART–SP-treated cells (Figure 4). Continuing the Cox-family investigation, *Cox-2* mRNA expression is decreased in SP-treated cells upon acute exposure (Figure 5). However, cells exposed to the HAART–SP treatment showed a significant decrease in *Cox-2* mRNA expression (Figure 5). SP has been proven to inhibit Cox-2 expression. In addition, SP exerts regulatory effects on mitogen-activated protein kinase (MAPK) activation pathways, such as c-Jun N-terminal kinase (JNK) [59,60,61]. The data indicate that *JNK* mRNA was reduced by SP, which agrees with previous studies. Moreso, SP and HAART showed synergy except TDF. Jun N-terminal kinases (JNK) belong to the superfamily of MAP kinases that are involved in the regulation of cell proliferation, differentiation, and apoptosis [46]. The *JNK* mRNA expression decreased in SP- and HAART-treated cells. HAART–SP-treated cells showed a decrease in the expression of *JNK* mRNA except TDF-SP (Figure 6).

Cox is a key enzyme for the conversion of arachidonic acid to prostaglandins and has two isozymes: Cox-1 and Cox-2. It has been found that overexpression of Cox-2 in cancer cell lines promotes their ability to invade surrounding tissues as well as increases cell invasion in gastric cancer. Some miRNAs downregulated the expression of *Cox-1* and *Cox-2* genes and thereby inhibited cell invasion [62]. This study investigated the expression of miRNAs that target Cox-1/2 mRNAs and evaluated the effect of SP on the expression of the *Cox-1/2* mRNAs in HepG2 cells. In the current study, miRNA and mRNA expression was performed to find the correlation in the expression of miRNAs (miR-146a and miR-155) and *Cox-1/2* mRNA [62]. The present study shows a significant reduction of *Cox-2*, this is an indication that SP might be targeting Cox-2 as one of the many mechanisms to inhibit inflammation.

Cox-1 is known to be present in most tissues that involve the maintenance of tissue homeostasis and cell signaling. Furthermore, Cox-1 is shown in angiogenesis in endothelial cells. Cox-2 is a well-known gene associated with inflammatory mediation and participates in numerous biological processes such as pain, inflammation, cancer, angiogenesis, carcinogenesis, or the development of immunity [63]. According to Cheng, Zhao, Ke, Wang, Cao, Liu, He and Rong [64], the inhibition of miR-155 and Cox-2 provides a protective effect in high-glucose conditions [64]. MiR-155 enhances Cox-2 expression and is an established regulator of epithelial–mesenchymal transition and inflammation [65]. Some natural compounds suppress inflammatory activity, especially those that are found in traditional medicine and dietary supplements, which have the potential to be developed as a Cox-2 inhibitor [63]. Cox-1 expression increased post-treatment HAART–SP in this present study and is a sign of a protective function and successful synergy between SP and HAART (Figure 4). This study found that SP potentially mitigates metabolic syndrome characteristics via the regulation of inflammatory miRNAs. We hereby recommend further exploration on the synergistic roles of SP and HAART in the expression of microRNA, with more focus on inflammatory pathway.

## 4. Materials and Methods

### 4.1. Materials

*Spirulina platensis*, extracted from *Spirulina platensis* (Shewal) capsules, were obtained from HeriCure Healthcare Ltd. (Pune, India); a 10 mg/mL aqueous stock solution of the extract was prepared from the capsule content (*Spirulina platensis* from the capsules was dissolved in distilled water (dH_2_O)), and the solution was filtered (0.45 μm) and used to prepare the concentrations of *Spirulina platensis* extract required for the study. The extract was then incubated at −80 °C for 24 h and lyophilized for 48 h using the Vis Tis sp Scientific freeze dryer (Warminster, Bucks County, PA, USA) (−46 °C, 79 mT,). The final weight of the extracts was obtained, and the extracts were stored in the dark at 4 °C until further use. Freeze drying is one of the finest treatment choices for heat-sensitive cyanobacteria, such as spirulina, as it results in the least number of alterations to their nutritional, sensory, and physicochemical properties, leaving the lyophilized products identical to fresh biomass [66]. Antiretroviral drugs were obtained from the NIH AIDS reagents program. The antiretroviral drug compounds were purchased from Pharmed Pharmaceuticals and extracted using dichloromethane, which was then removed using a standard laboratory rotary evaporator. The identity of the extracted compounds was confirmed using NMR analysis and showed a purity of >98%. The HepG2 cell line was acquired from Highveld Biologicals (Johannesburg, South Africa). Cell culture reagents and supplements were purchased from Lonza Bio-Whittaker (Basel, Switzerland) while all other reagents were purchased from Merck (Darmstadt, Germany).

### 4.2. Cell Culture

HepG2 cells were cultured in monolayer (10^6^ cells per 25 cm^3^ culture flask) with complete culture media (CCM: Eagle’s Essential Minimal Media (EMEM) supplemented with 10% foetal calf serum, 1% penstrepfungizone, and 1% L-glutamine) at 37 °C in a humidified incubator. Cells were allowed to reach 80% confluence in 25 cm^3^ flasks before treatment with only antiretrovirals (ARVs) using the plasma peak values from literature that represent the physiological concentrations of ARVs in humans (3TC: 6.6µM (1.51µg/mL), TDF: 0.3 µg/mL, FTC: 1.8 µg/mL) [67,68,69] in CCM for 96 h [70]. For the 96 h treatment, fresh cell culture medium containing ARVs treatment was replenished every 48 h. Thereafter, ARVs were removed, and cells were gently rinsed with 0.1 mol/L phosphate buffer saline (PBS) and treated with only 1.5 µg/mL SP on its own in CCM for 24 h. The 1.5 µg/mL SP concentration falls within the range that has been used in other studies [43], and MTT results supported this concentration. An untreated control, containing only CCM, was also prepared. Treatment for a 24 h time period was also conducted, containing only ARVs [71] and SP, separately.

### 4.3. 3-(4,5-Dimethylthiazol-2-yl)-2,5-Diphenyltetrazolium Bromide (MTT) Assay

The MTT assay was used to determine the cell viability. Cells (20,000 cells/well) were seeded in triplicate for each treatment in a 96-well microtiter plate and allowed to attach over a 24 h period (37 °C, 5% CO_2_). Thereafter, the treatment medium (SP) was added to the relevant wells from 0–5 µg/mL. After 24 h the treatment medium (SP) was removed and replaced with a solution containing 4 mg MTT salt, 800 μL PBS, and 4 mL CCM. The solution was incubated for 4 h and replaced with DMSO for 1 h. The absorbance was then read at 570 nm with a reference wavelength of 690 nm (BioTek μQuant spectrophotometer, Highland Park, Illinois, USA). The absorbance values were used to calculate the cell viability [72]. The log concentration and cell viability were analysed using GraphPad Prism 5 and Microsoft excel.

### 4.4. Reactive Oxygen Species Analyses

Intracellular ROS was quantified using the fluorometric 2′,7′-dichlorodihydrofluorescein-diacetate (H_2_DCF-DA) assay. Control and treated cells (50,000 cells per treatment) were incubated in 500 µL of 5 μmol/L H_2_DCF-DA stain (30 min, 37 °C). Thereafter, the stain was removed through centrifugation (400× *g*, 10 min, 24 °C) and cells were washed twice with 0.1 mol/L phosphate buffer saline (PBS). Cells were resuspended in 400 µL of 0.1 mol/L PBS and seeded in triplicate (100 µL/well) in a 96 well opaque microtiter plate. Fluorescence was measured with Modulus™ microplate luminometer (Turner Biosystems, Sunnyvale, CA, USA) using a blue filter with an excitation wavelength (λex) of 488 nm and emission wavelength (λem) of 529 nm. Results were expressed as relative fluorescence units (RFU).

### 4.5. Lactate Dehydrogenase (LDH) Activity

The LDH cytotoxicity detection kit (Roche, Mannheim, Germany) was used to measure cell death/damage. To measure LDH activity, supernatant (100 µL) was transferred into a 96-well microtitre plate in triplicate. Thereafter, substrate mixture (100 µL) containing catalyst (diaphorase/NAD^+^) and dye solution (INT/sodium lactate) was added to the supernatant and allowed to react at RT for 25 min. Optical density was measured at 500 nm (microplate reader—Bio-Tek µQuant). Results are presented as mean optical density.

### 4.6. Mitochondrial Membrane Potential

The mitochondrial membrane potential (Δψm) was measured by the JC-1 stain [73]. All samples, both control and treated cells (50,000 cells per treatment) were incubated in 200 µL of 5 µg/mL JC-1 stain (BD Biosciences, San Jose, NJ, USA) (20 min, 37 °C). The stain was removed via centrifugation (400× *g*, 10 min, 24 °C) and the cells were washed twice with JC-1 staining buffer. Cells were re-suspended in 400 µL of JC-1 staining buffer and seeded in an opaque 96-well plate in triplicate (100 µL/well). Fluorescence was quantified on a Modulus™ microplate reader (Turner Biosystems, Sunnyvale, CA). JC-1 monomers were measured with a blue filter (λex = 488 nm, λem = 529 nm) and JC-1 aggregates were measured with a green filter (λex = 524 nm, λem = 594 nm). The Δψm of the HepG2 cells was expressed as the fluorescence intensity ratio of JC-1 aggregates and JC-1 monomers [73].

### 4.7. Lipid Peroxidation Assessment

The thiobarbituric acid reactive substances (TBARS) assay measured lipid peroxidation by-products malondialdehyde (MDA) and other TBARS as a measure of oxidative damage to lipids. TBARS assay was conducted as per the method described by Abdul, Nagiah and Chuturgoon [74]. Absorbance of the samples was read using a spectrophotometer, λ = 532/600 nm. Results were expressed as MDA concentration (µM).

### 4.8. RNA Analysis

Total RNA was isolated according to the method described by Chuturgoon, Phulukdaree and Moodley [75]. Isolated RNA was quantified (Nanodrop 2000, Thermo Scientific, Waltham, MA, USA) and standardised to 1000 ng/μL. cDNA was synthesised from standardised RNA using the iScript cDNA synthesis kit (Bio-Rad). Thermocycler conditions for cDNA synthesis were 25 °C for 5 min, 42 °C for 30 min, 85 °C for 5 min, and a final hold at 4 °C. Gene expression was analysed using the SsoAdvanced™ Universal SYBR^®^ Green Supermix kit (Bio-Rad, Hercules, CA, USA). The mRNA expressions of Cox-1, Cox-2, Akt, and JNK were investigated using specific forward and reverse primers (Table 1). Reaction volumes that consisted of the following were prepared: SYBR green (5 μL), forward primer (1 μL), reverse primer (1 μL), nuclease-free water (2 μL), and cDNA template (1 μL). All reactions were carried out in triplicate. The samples were amplified using a CFX96 Touch™Real- Time PCR Detection System (Bio-Rad). The initial denaturation occurred at 95 °C (4 min). Thereafter, 37 cycles of denaturation (15 s, 95 °C), annealing (40 s; temperatures—Table 1), and extension (30 s, 72 °C) occurred. The method described by Livak and Schmittgen [76] was employed to determine the changes in relative mRNA expression, where 2^−ΔΔCt^ represents the fold change relative to the untreated control. The expression of the gene of interest was normalised against the housekeeping gene, Glyceraldehyde 3-phosphate dehydrogenase (GAPDH), which was amplified simultaneously under the same conditions.

### 4.9. Micro-RNA Analysis

The total RNA extracted (as previously described above) was reverse transcribed using the miScript ^®^ II RT Kit (Qiagen, Hilden, Germany; catalogue number 218160) as per manufacturer’s instructions. To quantify miRNA levels, miR-155 (MS00031486) and miR-146a (MS00033740) miScript Primer Assays were used, while RNU6 (MS00033740) was used as an internal control (Qiagen, Hilden, Germany). Experimental protocol was performed as per manufacturer’s instructions. The reaction was carried out with an initial activation step (95 °C, 15 min), followed by 40 cycles of denaturation (94 °C, 15 s), annealing (55 °C, 30 s), extension (70 °C, 30 s), and a plate read. Assays were conducted using CFX Touch™ Real Time PCR Detection System (Bio-Rad, Hercules, CA, USA). The analysis of data was conducted using the method described by Livak and Schmittgen (2^−ΔΔCT^) [76].

### 4.10. Statistical Analysis

GraphPad Prism version 5.0 (GraphPad Software Inc., San Diego, CA, USA) was used to perform all statistical analyses. The one-way analysis of variance (ANOVA) followed by a Bonferroni test for multiple group comparison (data are presented as 95% CI) was used to determine statistical significance. All results were represented as the mean ± standard deviation unless otherwise stated. A value of *p* ˂ 0.05 was considered statistically significant.

## 5. Conclusions

SP mitigates metabolic syndrome characteristics via the inhibition of miRNA that promotes inflammation. Moreover, HAART–SP promotes ROS balance, which is important for mitochondrial quality control. SP maintains intracellular balance, reduces excess ROS, protects mitochondrial potential, prevents necrotic cell death, and enhances mitochondrial quality. Most of these SP qualities worked most favourably with HAART. We hereby recommend further investigation of SP’s ability to inhibit chronic negative effects of highly active antiretroviral therapy (HAART) in vitro via gene knockouts.

## Figures and Tables

**Table 1:** The annealing temperatures and primer sequences for the genes of interest.

Gene	Annealing Temperature	Primer	Sequence
*Cox-1*	50 °C	Forward Reverse	5′-CGCCAGTGAATCCCTGTTGTT-3′
			5′-AAGGTGGCATTGACAAACTCC-3′
*Cox-2*	53 °C	Forward Reverse	5′-TAAGTGCGATTGTACCCGGAC-3′
			5′-TTTGTAGCCATAGTCAGCATTGT-3′
*JNK*	59.7 °C	Forward Reverse	5′-GACGCCTTATGTAGTGACTCGC-3′
			5′-TCCTGGAAAGAGGATTTTGTGGC-3′
*GAPDH*		Forward Reverse	5′-TCCACCACCCTGTTGCTGTA-3′
			5′-ACCACAGTCCATGCCATCAC-3′

## Data Availability

Not applicable.
